# A pilot-scale floating closed culture system for the multicellular cyanobacterium *Arthrospira platensis* NIES-39

**DOI:** 10.1007/s10811-014-0484-2

**Published:** 2014-12-08

**Authors:** Masakazu Toyoshima, Shimpei Aikawa, Takahiro Yamagishi, Akihiko Kondo, Hiroshi Kawai

**Affiliations:** 1 Kobe University Research Center for Inland Seas Rokkodai, Nadaku, Kobe, 657-8501 Japan; 2 Department of Chemical Science and Engineering, Graduate School of Engineering, Kobe University, 1-1 Rokkodai, Nadaku, Kobe, 657-8501 Japan; 3 Core Research for Evolutional Science and Technology, Japan Science and Technology Agency, 3-5 Sanbancho, Chiyodaku, Tokyo, 102-0075 Japan

**Keywords:** *Arthrospira* (*Spirulina*) *platensis* NIES-39, Biomass productivity, Floating closed culture system, Modified seawater medium, Outdoor culture

## Abstract

Microalgae are considered to be efficient bio-resources for biofuels and bio-based chemicals because they generally have high productivity. The filamentous cyanobacterium *Arthrospira* (*Spirulina*) *platensis* has been widely used for food, feed, and nutrient supplements and is usually cultivated in open ponds. In order to extend the surface area for growing this alga, we designed a pilot-scale floating closed culture system for cultivating *A. platensis* on open water and compared the growth and quality of the alga harvested at both subtropical and temperate regions. The biomass productivity of *A. platensis* NIES-39 was ca. 9 g dry biomass m^−2^ day^−1^ in summer at Awaji Island (warm temperature region) and ca. 10 and 6 g dry biomass m^−2^ day^−1^ in autumn and winter, respectively, at Ishigaki Island, (subtropical region) in Japan. If seawater can be used for culture media, culture cost can be reduced; therefore, we examined the influence of seawater salt concentrations on the growth of *A. platensis* NIES-39. Growth rates of *A. platensis* NIES-39 in diluted seawater with enrichment of 2.5 g L^−1^ NaNO3, 0.01 g L^−1^ FeSO_4_·7H_2_O, and 0.08 g L^−1^ Na_2_EDTA were considerably lower than SOT medium, but the biomass productivity (dry weight) was comparable to SOT medium. This is explained by the heavier cell weight of the alga grown in modified seawater media compared to the alga grown in SOT medium. Furthermore, *A. platensis* grown in modified seawater-based medium exhibited self-flocculation and had more loosely coiled trichomes.

## Introduction

Development of clean and sustainable biofuels has gained significant support owing to global climate change, the shortage of energy, and petroleum supplies. Photosynthetic microorganisms, including eukaryotic microalgae and prokaryotic cyanobacteria, are considered to be generally more efficient than land plants in converting solar energy and recycling CO_2_ into fuels (Dismukes et al. 2008). In order to avoid conflict with the food industry for arable land, it is desirable to use sites where agricultural use is difficult (e.g., desert, ocean surface, etc.). However, in order to achieve large-scale commercial uses of photosynthetic microorganisms, it is important to reduce the production cost by developing low-cost culture facilities and culture media and efficient harvesting methods. Strains of the cyanobacterium *Arthrospira* (*Spirulina*) *platensis*, originally isolated from an African alkaline lake, have seen wide commercial use because they grow vigorously in tropical and subtropical climatic conditions (Kim et al. 2007; Lodi et al. 2005; Ogbonda et al. 2007) and have high tolerance to alkaline and high salt concentrations (Zeng and Vonshak 1998). *A. platensis* has been widely used for foods, feeds, and nutrient supplements (Anupama and Ravindra 2000; Spolaore et al. 2006) owing to its high nutritional value (Converti et al. 2006). Furthermore, it has great potential as a source of bioenergy, because its photosynthetic storage product (i.e., glycogen) is an effective substrate for alcohol fermentation (Aikawa et al. 2012, 2013).

The whole-genome DNA sequence of *A. platensis* NIES-39 has been determined (Fujisawa et al. 2010). On the basis of this genome sequence, gene manipulation through genetic engineering is anticipated to improve growth performance and produce useful bio-products.

Open ponds and raceway methods have been widely used for the mass culture of photosynthetic microorganisms, but only a few species can be stably maintained in such systems, because diverse contaminating organisms (e.g., algae, bacteria, fungi, protozoa) reduce production of the targeted alga. To avoid contamination, species that can grow under extreme conditions (e.g., high temperature, alkaline or acidic conditions, high salt concentrations, etc.) that inhibit growth of contaminants have been selected for cultures in open systems. *A. platensis* is one of the most widely cultured commercial photosynthetic microorganisms in traditional open systems, because of its preference for alkaline conditions (Vonshak and Richmond 1988; Lu et al. 2011) that do not allow the growth of most contaminants.

The growth and biomass productivity of *A. platensis* depend on several factors such as solar irradiance in the photosynthetically active range (PAR), pH, quantities of contaminants, temperature, dissolved oxygen concentration, salinity, amount of agitation or aeration, and nutrient availability (Colla et al. 2004; Ogbonna et al. 1995; Vonshak 1997). In the traditional open culture systems of *A. platensis*, light intensity, temperature, and dissolved oxygen concentration have been considered to be most important for determining growth (Chaumount 1993; Borowitzka and Moheimani 2013).

For use of transformed strains of *A. platensis* in larger-scale cultures, it is essential to establish a closed culture system to avoid the risk of the strains escaping to natural habitats. Fully closed photobioreactors also allow better control of the culture conditions than open systems. However, closed photobioreactors generally cost more than open systems, due to higher construction, operation, and maintenance costs. The production cost is especially important when the price of the products is not high, as in the case of bioenergy production. Also, both traditional open systems and fully closed photobioreactors require land for their operation. Considering the abovementioned aspects, we aimed to establish a low-cost, continuous/semicontinuous, closed culture system using open water surfaces.

*A. platensis* is a planktonic multicellular cyanobacterium with helical filamentous form. It has relatively large individual cells of 6–16 μm in diameter, 30–70 μm in helix diameter, 12–72 μm helix pitch (Pelosi et al. 1982), and strong motility by the rotation of the helical trichome. These characters are beneficial in mass cultures because the trichomes tend to remain suspended in the culture media and are rather easy to harvest using fine mesh screens. Energy and costs for pretreatment of the harvested algae (e.g., drying, cell breakage, extraction, etc.) are important elements of the production cost. However, in *A. platensis*, the harvested cells can be used for fermentation processes without pretreatment or enzymatic hydrolysis (Aikawa et al. 2013). For mass cultures, use of the sea surface of subtropical/tropical coasts or open oceans provides high solar irradiances and stable growth temperatures, because the temperature range of the tropical surface water (20–30 °C) matches the optimum growth range of this species. *A. platensis* is usually cultured utilizing a chemically defined medium, and a significant portion of the production cost is contributed by these chemicals. Hence, the use of low-cost media such as seawater media will reduce the production costs. In studies using seawater as an alternative medium, pretreatment to remove Ca^2+^ and Mg^2+^ (Faucher et al. 1979; Leema et al. 2010) and supplementation with specific nutrients have been employed under laboratory conditions (Materassi et al. 1984) or in outdoor raceways (Tredici et al. 1986; Wu et al. 1993).

Here we discuss the design and operation of a pilot-scale floating closed culture system and the use of low-cost media (modified SOT media and modified seawater media) for the continuous culture of *A. platensis* using outdoor water surfaces such as the sea and ponds.

## Materials and methods

### Strain and laboratory culture

The axenic culture strain *Arthrospira platensis* NIES-39 (National Institute for Environmental Studies) was used for all experiments. For batch cultures, the alga was grown and maintained at 30 °C in 16 L modified SOT medium (Aikawa et al. 2012) in 20 L polycarbonate containers (Nalgene Clearboy; USA) under continuous illumination provided by LED lamps (ECL-HPL60; Ecorica Inc., Japan) with aeration. Because a linear correlation was seen between the optical density at 750 nm (OD_750_) and the dry weight of the harvested alga, cell density was estimated by measuring OD_750_ (Watanabe et al. 2001). The optical density was measured using a DU 730 Spectrophotometer (Beckman Coulter, USA). Morphology of the trichomes (helical filaments) under different growth conditions was examined using a BX51 microscope (Olympus, Japan) equipped with a DP21 digital camera. The helix pitch of *A. platensis* NIES-39 was measured based on digital images using ImageJ 1.38x software (http://rsb.info.nih.gov/ij/).

**Fig. 1:**
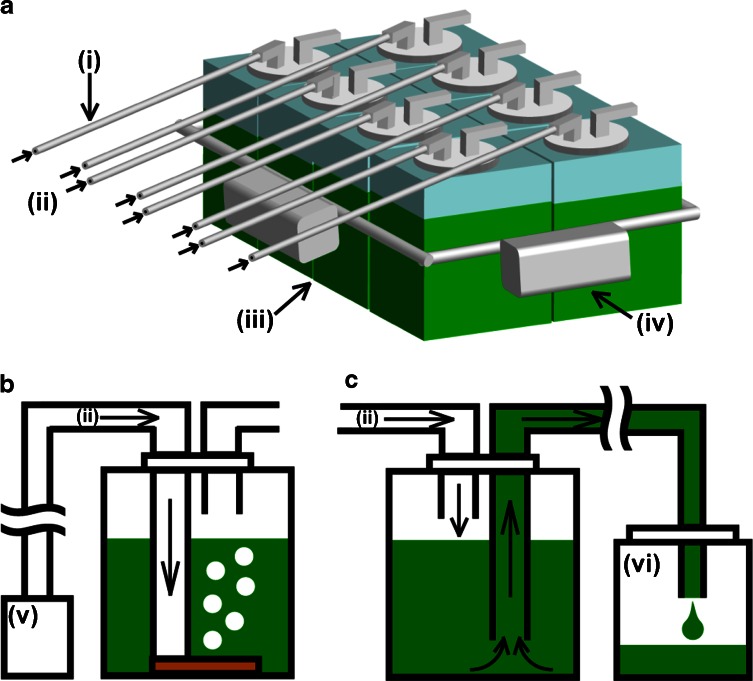
Schematic diagram of the floating closed culture system: **a** floating closed culture system, **b** culture system during culture, and **c** harvest of *A. platensis* NIES-39 biomass. (*i*) silicone tube, (*ii*) air, (*iii*) polypropylene or polycarbonate container, (*iv*) float, (*v*) air pump, (*vi*) harvest tank

The floating closed culture system is shown in Fig. 1a. It consists of 10 (0.22 × 0.22 × 0.22 m; top surface area of 0.048 m^2^) and 20 L (0.27 × 0.27 × 0.27 m; 0.073 m^2^) transparent polyethylene (PE) culture containers (Baron Box, Sansyo Co., Tokyo, Japan) or 20 L (0.32 × 0.22 × 0.38 m; 0.070 m^2^) transparent polycarbonate (PC) containers (Clearboy, Nalgene). Six, eight, or nine culture containers were bound into a raft, to which several air floats were attached for floatation. The 10 and 20 L containers were filled with 8 and 16 L culture medium, respectively, allowing airspace over the surface of the medium to permit escape of aeration air through an exit port. The cultures were inoculated with 3–4 % (*v*/*v*) of stationary-stage inocula under aseptic conditions. Compressed air was supplied to the bottom of each culture container using a porous air diffuser at a flow rate of approximately 3 L min^−1^.

### Harvest and biomass productivity quantitation

The method for harvesting cultures from the floating culture container is shown in Fig. 1b. The air exit port of the tank was closed, and the air pressure pushed the culture out through a silicone rubber tube to a harvest tank on shore. Harvested cultures were concentrated using nylon filter screens of 32 μm mesh. The harvested algae were dried at 90 °C for 48 h, and the dry weight was measured.

For the analysis of glycogen, the collected biomass was frozen in liquid nitrogen, dried using a freeze-dryer, and stored at −80 °C until sample extraction was performed. Glycogen was extracted from the cells by the method of Aikawa et al. (2012). Glycogen contents were determined by a LC Prominence high-performance liquid chromatograph (HPLC) (Shimadzu, Japan) using an OHpak SB-806M HQ size exclusion HPLC column (Shodex, Japan) and a RID-10A refractive index detector (Shimadzu) as previously described (Izumi et al. 2013).

### Floating closed culture system

**Fig. 2:**
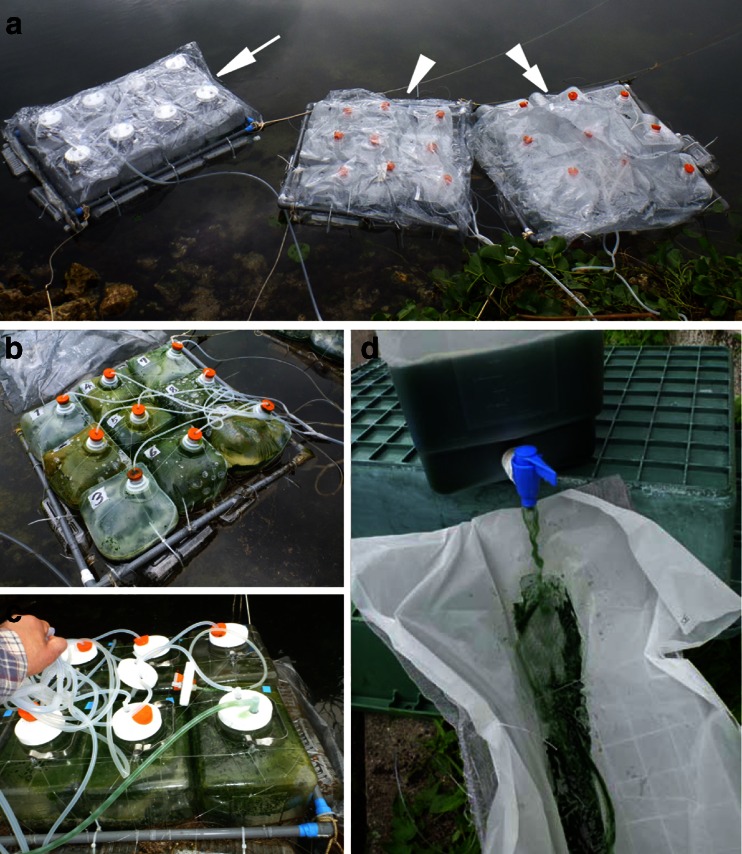
Culture of *A. platensis* NIES-39 using floating closed culture system at Ishigaki Island. **a** Floating system consists of PE containers with volume of 10 (*arrowhead*) and 20 L (*double arrowhead*) or PC containers with volume of 20 L (*arrow*). **b** 10 L PE container raft after culture. **c** 20 L PC containers after culture. **d** Continuous filtration using nylon mesh screen

In order to compare the growth and biomass production at different climatic (latitude) sites, field experiments were carried out using the experimental ponds at the marine laboratory of Kobe University Research Center for Inland Seas at Awaji Island (warm temperate region, 34° 34′ 55″ N, 135° 01′ 21″ E) and Seikai National Fisheries Research Institute of Fisheries Research Agency (FRA) at Ishigaki Island (subtropical region, 24° 20′ 04″ N, 124° 09′ 22″ E) (Fig. 2). The water temperatures of the ponds were recorded using a TidbiT v2 temperature data logger (Onset Computer Corp., USA) every 10 min. Modified SOT and 1/4 SOT [fourfold diluted (*v*/*v*) SOT medium] media were used in separate containers. The outdoor experimental periods were operated from 26 August to 15 September in 2011 (period A1, 10 L PE containers) at Awaji Island, from 9 September to 6 October in 2011 (period I1, 10 and 20 L PE containers), and from 1 February to 12 March in 2012 (period I2, 20 L PC containers added to the culture of period I1) at Ishigaki Island. Airflow rate to each culture container was 3 L min^−1^. The global solar radiation data (MJ m^−2^) published by the Japan Meteorological Agency (JMA) was referenced for Awaji Island and Ishigaki Island.

**Fig. 3:**
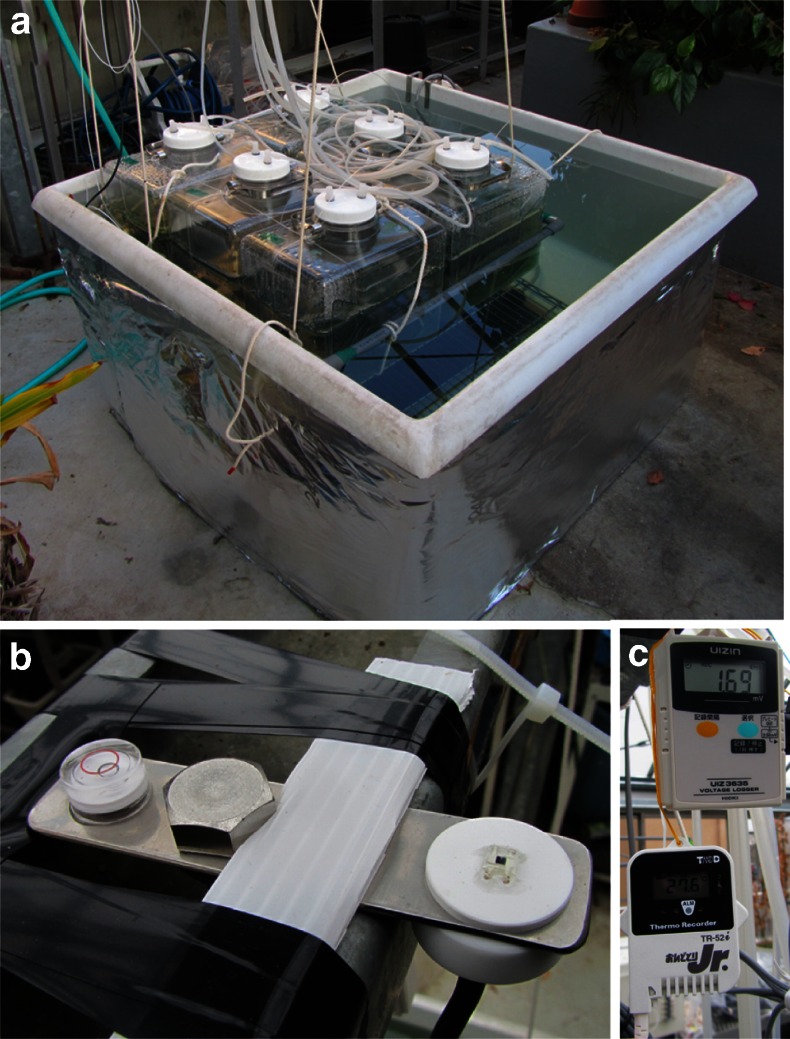
Culture of *A. platensis* NIES-39 in greenhouse. **a** The floating culture system in a water bath in greenhouse. **b** Light quantum meter. **c** Temperature data logger

These outdoor experiments were supplemented by indoor experiments carried out in a glass greenhouse in the Faculty of Science, Kobe University (34° 43′ 31″ N, 135° 14′ 08″ E) (Fig. 3). In order to compare the growth and biomass produced under a range of salt concentrations, four different seawater-based media of different seawater ratios were used. Diluted seawater media of 1/2 seawater (1/2 SW; 1 part filtered seawater: 1 part deionized water, *v*/*v*), 1/3 SW (1 part filtered seawater: 2 parts deionized water, *v*/*v*), 1/4 SW (1 part filtered seawater: 3 parts deionized water, *v*/*v*), and 1/5 SW (1 part filtered seawater: 4 parts deionized water, *v*/*v*) were used with addition 2.5 g L^−1^ NaNO_3_, 0.01 g L^−1^ FeSO_4_·7H_2_O, and 0.08 g L^−1^ Na_2_EDTA. The seawater was obtained from Osaka Bay, Hyogo, Japan (salinity 31.5‰, pH 7.8). The seawater was not pretreated to remove Ca^2+^ and Mg^2+^, unlike Faucher et al. (1979) and Leema et al. (2010).

The raft consisting of six 20 L PC containers was floated on a 1 m × 1 m heated water bath in the greenhouse. Airflow rate was 2.5 L min^−1^. The temperature of the water bath was regulated at 28 °C by a thermostat, and the water temperature was measured every 10 min using a TR-51i internal temperature data logger (T & D Co., Japan). The photosynthetic photon flux density (PPFD) was measured every 10 min using a UIZ-PAR light quantum meter (Uizin, Japan). The pH of the culture media was measured using a F-55 pH/Water Quality Analyzer (Horiba, Japan).

## Results

### Growth, harvested biomass, and culture condition in outdoor cultures

**Fig. 4:**
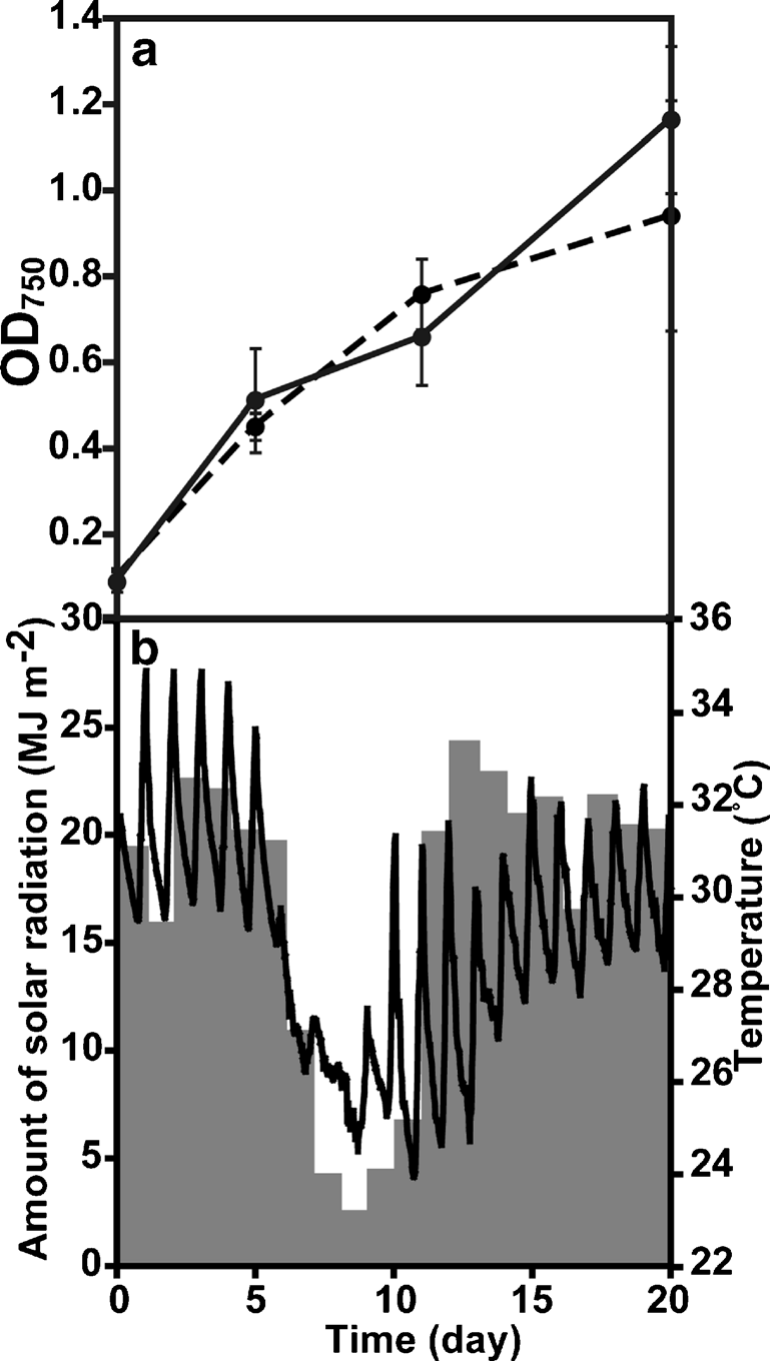
Growth of *A. platensis* NIES-39 at Awaji Island in summer. **a** Growth of *A. platensis* NIES-39 using the floating closed culture system. *Solid lines* indicate SOT and *broken lines* 1/4 SOT. **b** Temperature (*lines*) and the amount of solar radiation (*bars*). *Error bars* show S. D.

**Fig. 5:**
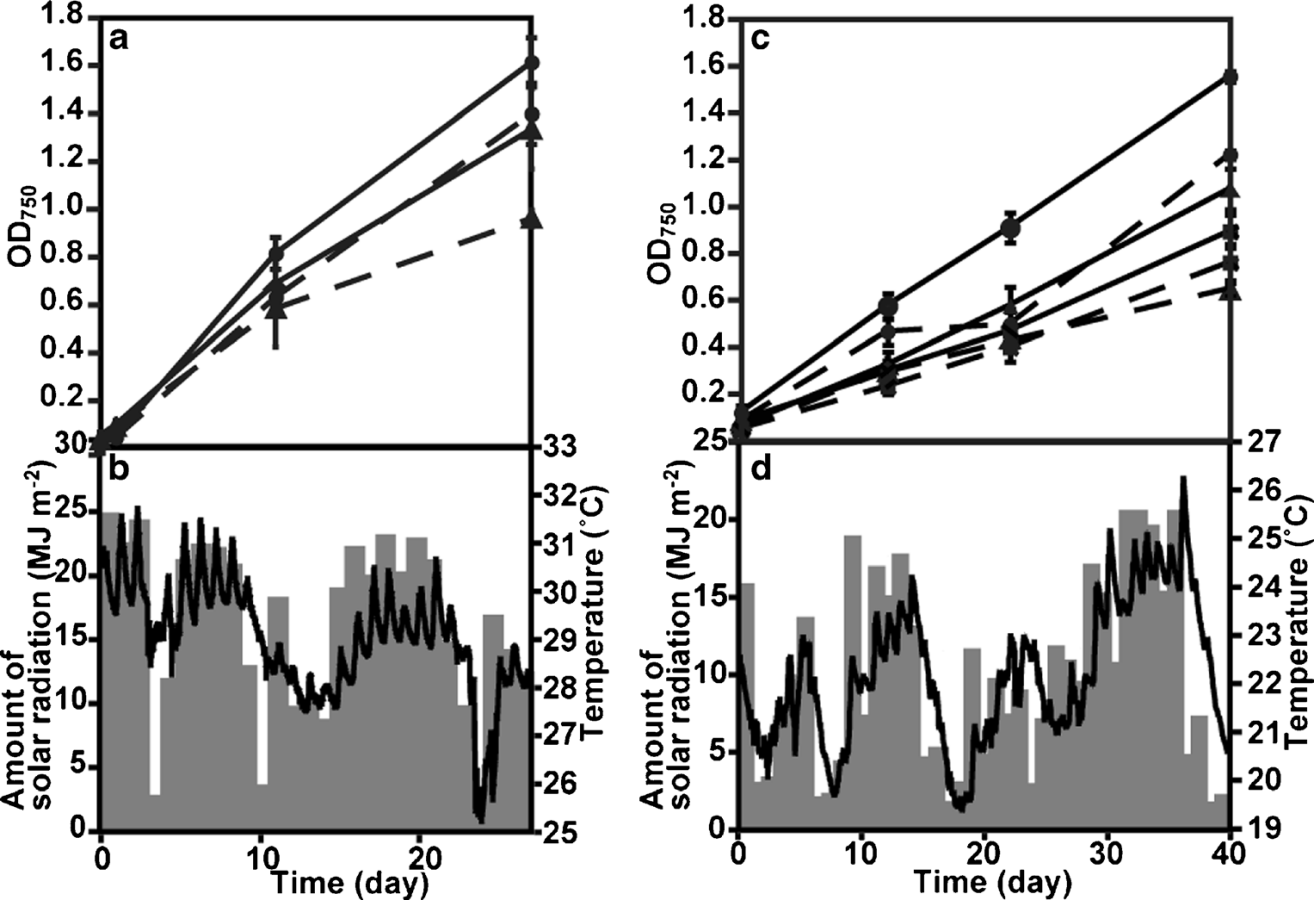
Growth of *A. platensis* NIES-39 at Ishigaki Island. **a**, **b** Results of period I1. **c**, **d** Results of period I2. *Panels* **a** and **c** show the growth of *A. platensis* NIES-39 using the floating closed culture system. SOT is indicated by *solid lines*, 1/4 SOT *broken lines*, 10 L PE containers *circles*, 20 L PE containers *triangles*, and 20 L PC containers *squares. Panels* **b** and **d** show the temperature (*lines*) and the amount of solar radiation (*bars*). *Error bars* show S. D.

In the culture experiments using SOT medium in 10 and 20 L PE containers, the biomass productivity of *A. platensis* NIES-39 was 8.71 ± 1.79 g dry biomass m^−2^ day^−1^ (10 L) in period A1 (summer, 20 days, average solar radiation was 16.9 MJ m^−2^ day^−1^, average temperature was 29.0 °C) at Awaji Island, and 9.46 ± 0.45 g dry biomass m^−2^ day^−1^ (10 L) and 9.76 ± 1.27 g dry biomass m^−2^ day^−1^ (20 L) in period I1 (autumn, 27 days, 16.2 MJ m^−2^ day^−1^, 27.0 °C) at Ishigaki Island (Figs. 4 and 5a, b and Table 1). During period I2 (winter, 40 days, 9.8 MJ m^−2^ day^−1^, 22.2 °C), the biomass productivity was 5.78 ± 0.08 g dry biomass m^−2^ day^−1^ (10 L), 5.30 ± 0.43 g dry biomass m^−2^ day^−1^ (20 L), and 4.57 ± 0.36 g dry biomass m^−2^ day^−1^ (20 L PC containers) (Fig. 5c, d and Table 1).

**Table 1:** Biomass productivity of *A. platensis* NIES-39 cultured at Awaji Island and Ishigaki Island

Period	Solar radiation (MJ m^−2^ day^−1^)	Min./Max./Ave. temperature (°C)	Volume (L)	Medium	Final OD_750_	Dry biomass (g L^−1^)	Biomass productivity (g dry biomass m^−2^ day^−1^)
A1	16.9	23.9/34.9/29.0	10	SOT	1.16 ± 0.17	1.05 ± 0.22	8.71 ± 1.79
				1/4 SOT	0.94 ± 0.27	0.70 ± 0.07	5.82 ± 0.59
I1	16.2	25.3/31.7/27.0	10	SOT	1.61 ± 0.10	1.55 ± 0.07	9.46 ± 0.45
			10	1/4 SOT	1.40 ± 0.13	1.23 ± 0.13	7.50 ± 0.82
			20	SOT	1.33 ± 0.17	1.20 ± 0.16	9.76 ± 1.27
			20	1/4 SOT	0.96 ± 0.04	0.83 ± 0.06	6.77 ± 0.48
I2	9.8	19.3/26.2/22.2	10	SOT	1.55 ± 0.02	1.40 ± 0.02	5.78 ± 0.08
			10	1/4 SOT	1.22 ± 0.02	1.10 ± 0.02	4.54 ± 0.06
			20	SOT	1.07 ± 0.09	0.97 ± 0.08	5.30 ± 0.43
			20	1/4 SOT	0.65 ± 0.03	0.58 ± 0.02	3.19 ± 0.13
			20^a^	SOT	0.89 ± 0.07	0.80 ± 0.06	4.57 ± 0.36
			20^a^	1/4 SOT	0.76 ± 0.08	0.68 ± 0.07	3.89 ± 0.40

*PPFD* photosynthetic photon flux density

^a^The results from culture in PC containers

### Growth using modified seawater-based media under sunlight

**Fig. 6:**
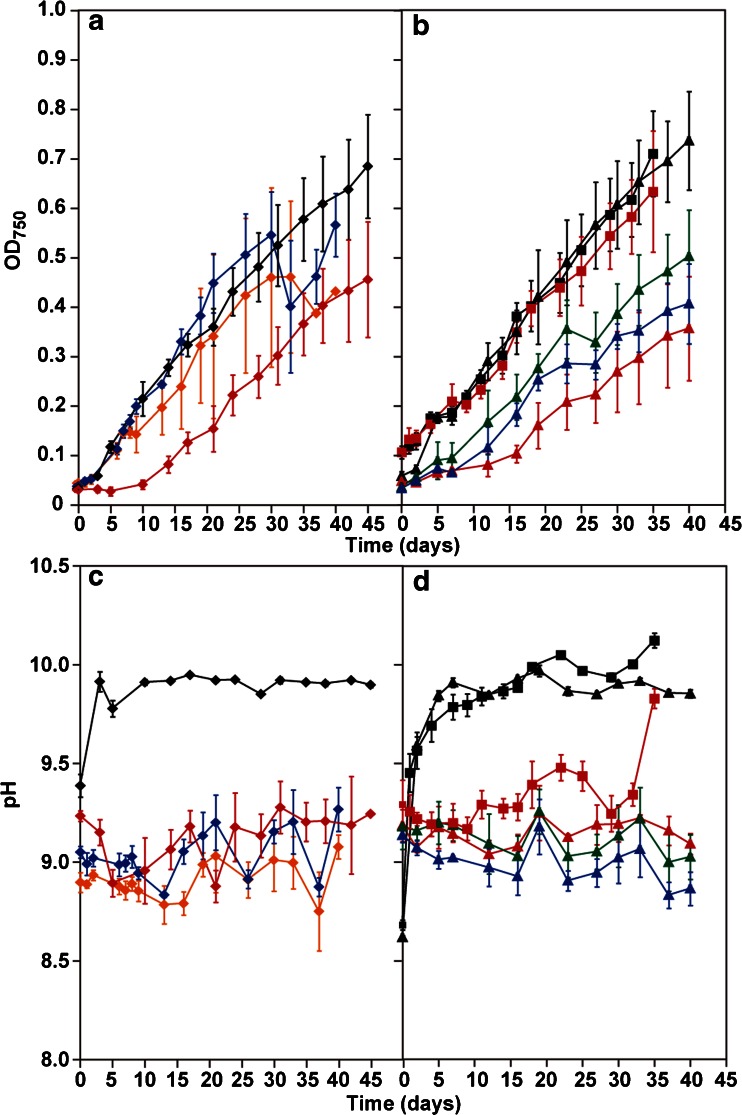
Growth of *A. platensis* NIES-39 and changes of pH in cultures using modified seawater medium. **a**, **b** Growth of *A. platensis* NIES-39 in 1/2, 1/3, 1/4, 1/5 SW, and SOT media in greenhouse. **c**, **d** Changes of pH of *A. platensis* NIES-39 greenhouse cultures in 1/2, 1/3, 1/4, and 1/5 SW, and SOT media. In the cultures from 26 August to 30 September, 2013 (35 days), *A. platensis* NIES-39 was grown in SOT (*black lines and squares*) and 1/4 SW medium (*red lines and squares*). In the cultures from 9 October to 18 November, 2013 (40 days), SOT is indicated by *black lines and triangles*, 1/4 SW medium *red lines and triangles*, 1/3 SW medium *blue lines and triangles*, and 1/5 SW medium *green lines and triangles*. In the cultures from 8 January to 17 February, 2014 (40 days), 1/2 SW medium is indicated by *orange lines and diamonds* and 1/3 SW medium *blue lines and diamonds*. In the cultures from 24 January to 10 March, 2014 (45 days), SOT is indicated by *black lines and diamonds* and 1/4 SW medium *red lines and diamonds. Error bars* show S. D.

In the cultures from 26 August to 30 September, 2013 (35 days), *A. platensis* NIES-39 was grown in SOT and 1/4 SW media. The biomass productivities were 4.09 ± 0.47 g dry biomass m^−2^ day^−1^ in SOT medium and 5.40 ± 1.16 g dry biomass m^−2^ day^−1^ in 1/4 SW medium (Fig. 6a and Table 2).

**Table 2:** Biomass productivity of *A. platensis* NIES-39 cultured using modified seawater media

Period	PPFD [Max./Ave.] (μmol photons m^−2^ s^−1^)	Temperature [Min./Max./Ave.] (°C)	Medium	Final OD_750_	Dry weight (g L^−1^)	Biomass productivity (g dry biomass m^−2^ day^−1^)	Glycogen content (% of dry biomass)
2013, 26 Aug–30 Sept	1486/135	27.4/30.2/28.0	SOT	0.709 ± 0.086	0.63 ± 0.07	4.09 ± 0.47	
			1/4 SW	0.633 ± 0.123	0.83 ± 0.18	5.40 ± 1.16	
2013, 9 Oct–18 Nov	1310/95	18.3/31.6/27.3	SOT	0.736 ± 0.099	1.40 ± 0.10	3.95 ± 0.58	
			1/4 SW	0.356 ± 0.105	1.15 ± 0.05	3.23 ± 0.28	
	1331/82	26.8/29.0/27.8	1/3 SW	0.406 ± 0.081	1.07 ± 0.15	3.00 ± 0.84	
			1/5 SW	0.502 ± 0.093	1.13 ± 0.10	3.18 ± 0.54	
2014, 8 Jan–17 Feb	1084/96	24.3/28.2/27.6	1/2 SW	0.432 ± 0.079	0.78 ± 0.27	4.42 ± 1.56	13.3 ± 3.0
			1/3 SW	0.566 ± 0.064	0.90 ± 0.24	5.11 ± 1.36	14.0 ± 2.8
2014, 24 Jan–10 Mar	1065/89	25.1/28.4/27.7	SOT	0.685 ± 0.105	0.65 ± 0.08	3.29 ± 0.43	5.8 ± 1.6
			1/4 SW	0.456 ± 0.117	0.40 ± 0.21	2.00 ± 1.05	12.1 ± 1.7

In the cultures from 9 October to 18 November, 2013 (40 days), *A. platensis* NIES-39 was grown in SOT and 1/4 SW media in the water bath simultaneously. Also, *A. platensis* NIES-39 was grown in 1/3 and 1/5 SW media in the water bath simultaneously. The biomass productivities were 3.95 ± 0.58, 3.00 ± 0.84, 3.23 ± 0.28, and 3.18 ± 0.54 g dry biomass m^−2^ day^−1^ in SOT, 1/3, 1/4, and 1/5 SW media, respectively (Fig. 6a and Table 2).

In the cultures from 8 January to 17 February, 2014 (40 days), *A. platensis* NIES-39 was grown in 1/2 and 1/3 SW media. The biomass productivities were 4.42 ± 1.56 g dry biomass m^−2^ day^−1^ in 1/2 SW medium, and 5.11 ± 1.36 g dry biomass m^−2^ day^−1^ in 1/3 SW medium. Glycogen content of *A. platensis* NIES-39 was 13.3 ± 3.0 and 14.0 ± 2.8 % grown in 1/2 and 1/3 SW media, respectively (Fig. 6a and Table 2).

In the cultures from 24 January to 10 March, 2014 (45 days), *A. platensis* NIES-39 was grown in SOT and 1/4 SW media. The biomass productivities were 3.29 ± 0.43 g dry biomass m^−2^ day^−1^ in SOT medium and 2.00 ± 1.05 g dry biomass m^−2^ day^−1^ in 1/4 SW medium. Glycogen content of *A. platensis* NIES-39 was 5.8 ± 1.6 and 12.1 ± 1.7 % grown in SOT and 1/4 SW media, respectively (Fig. 6a and Table 2).

### Self-flocculation and morphological changes of *A. platensis* NIES-39 grown in modified seawater-based media

**Fig. 7:**
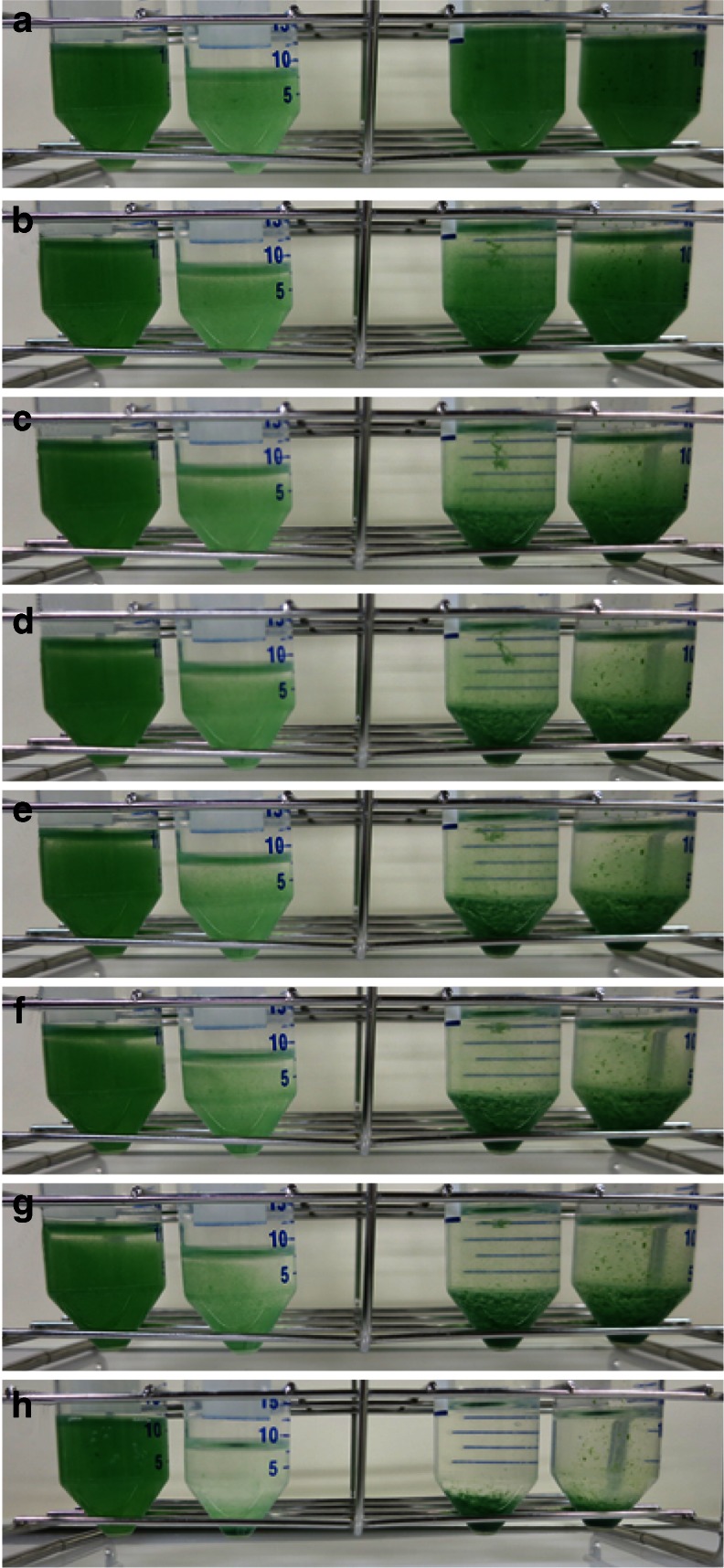
Self-flocculation of *A. platensis* NIES-39 cultured in modified seawater media. *A. platensis* NIES-39 grown in SOT, 1/4, 1/3, 1/2 SW media from the left tube. The self-flocculation of cultures was monitored by taking photographs at 0 (**a**), 15 (**b**), 30 (**c**), 45 (**d**), 60 (**e**), 90 (**f**), 120 min (**g**), and 24 h (**h**)

**Fig. 8:**
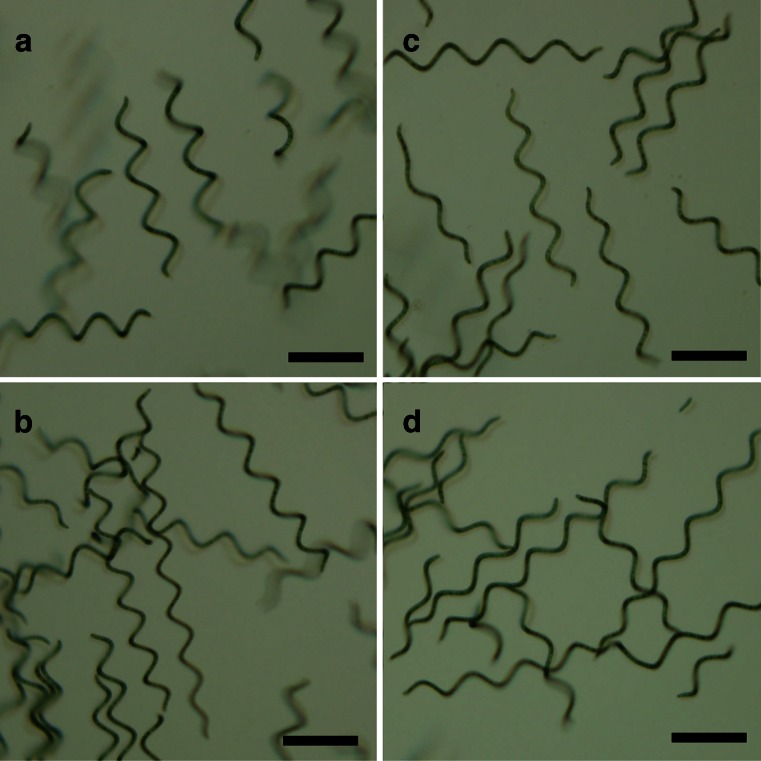
Helical form of *A. platensis* NIES-39 grown in SOT and modified seawater media. **a** SOT medium. **b** 1/4 SW medium. **c** 1/3 SW medium. **d** 1/2 SW medium. *Bars* = 100 μm

As shown in Fig. 7 in modified seawater-based media, the filaments aggregated and soon became flocculated. The helix pitches of the trichomes cultured in modified seawater-based media were 68.1 ± 5.8, 65.4 ± 6.2, 72.5 ± 4.1, and 75.8 ± 5.0 μm grown in SOT, 1/4, 1/3, and 1/2 SW media, respectively (Fig. 8).

## Discussion

Various types of integrated culture systems for microalgae have been proposed (Singh and Sharma 2012; Wang et al. 2012). In the present study, we demonstrated that *A. platensis* could be effectively cultivated using a floating culture system using only aeration as a power source for growth and harvest. The PE and PC containers gave comparable growth of *A. platensis*. However, for the harvesting process of the cultures using air pressure, PC containers were more suitable, because the walls of the PE containers tended to expand under high air pressure, requiring harvesting under lower air pressure for a longer time.

The biomass productivities of *A. platensis* in the warm temperature habitat were ca. 9 g dry biomass m^−2^ day^−1^ in summer (period A1) and in the subtropical habitat were ca. 10 g dry biomass m^−2^ day^−1^ in autumn (period I1) and ca. 6 g dry biomass m^−2^ day^−1^ in winter (period I2) (Table 1). The 20 L cultures showed higher productivity than 10 L in autumn, and 10 L cultures showed higher productivity than 20 L in winter. Thus, in high intensity solar radiation, biomass productivity is not reduced using deeper culture containers. These results were comparable to those reported for open ponds by the *Spirulina* industry in Inner Mongolia, China (Lu et al. 2011) and Southern Spain (Jiménez et al. 2003). Our biomass productivity was as good as that reported for tubular bubble-column reactors in Southern Italy during autumn and winter (Chini-Zitelli et al. 1996). On the other hand, our results were similar to the results with other microalgae: cultivation of *Chlorella vulgaris* using raceways and photobioreactors in the UK (Stephenson et al. 2010), cultivation of *Nannochloropsis* sp. using raceways and photobioreactors (Jorquera et al. 2010), and mixotrophic cultivation of *Chlamydomonas globosa*, *Chlorella minutissima*, and *Scenedesmus bijuga* using vertical tank reactors and raceways in greenhouse in the USA (Chinnasamy et al. 2010). Furthermore, if we would harvest *A. platensis* at the beginning of stationary phase, higher biomass productivity might be obtained, because our results were based on harvesting at late stationary phase. Actually, based on the period I1 experiment using 10 L PP containers, we can project that the biomass productivity of *A. platensis* becomes ca. 12 g dry biomass m^−2^ day^−1^ if we harvested cells at 11 days after inoculation (the biomass productivity was ca. 9.5 g dry biomass m^−2^ day^−1^ when we harvested at 27 days). The equipment costs required for this culture system are only the costs of the ventilation pump and the culture containers.

The costs per 1 m^2^ of 10 L polyethylene (PE), 20 L PE, and 20 L polycarbonate (PC) containers were ca. 66, 60, and US$4000, respectively. Both PE and PC containers gave good results for the growth of the cultures, but considering the high air pressure required for harvesting, PC was more suitable for the process. PC containers are rather expensive, but polyethylene terephthalate (PET) containers are considered to have sufficient transparency and strength comparable to PC, and the cost is comparable to that of PE. In the present experiments, we did not use PET containers because there were no commercially available containers of this size, but for the practical development of the mass culture system, we suggest the use of PET containers.

Our floating culture system has a number of advantages compared to land-based algae cultivation systems. These floating culture systems are surrounded by seawater, a heat sink to prevent overheating [a major photobioreactor problem on land (Carvalho et al. 2006)]. Furthermore, using the ocean surface avoids conflict with the food industry for arable land and provides sufficient light even in waters with poor transparency. However, if culture systems float offshore, we must consider the influence of biofouling on algal productivity (Harris et al. 2013). Biofouling inevitably occurs on any exposed surface in the marine environment (Durr and Thomason 2010). Biofouling causes decreased buoyancy and accelerated degradation or corrosion of structures, along with impaired functions and significant costs associated with equipment maintenance, repair, or replacement (Edyyean 2010; Schultz et al. 2010). Furthermore, biofouling can influence both the quantity and quality of light that penetrates transparent photobioreactor materials (Brush and Nixon 2002; Wong et al. 2011). Such problems have particularly affected photobioreactors such as the Offshore Membrane Enclosures for Growing Algae (OMEGA) system, which is a photobioreactor consisting of large flexible plastic tubes (Harris et al. 2013). However, because the upper surface of the culture floats above the ocean surface, we believe that there will be very little light-blocking biofouling. Also, because we can adjust the buoyancy of our culture system by changing the volume of the culture medium, we can regulate how much of the culture container projects above the surface of the ocean surface. In fact, in our outdoor culture experiment, biofouling did not occur on the upper exposed surface of our culture containers. Even in cases of replacing the culture containers, the cost of PET containers is reasonably low.

The temperature of the ocean surface around Awaji Island was 22.9–29.3 °C (av. 26.2 °C), and Ishigaki Island was 27.0–30.4 °C (av. 28.5 °C), and 21.4–23.7 °C (av. 22.6 °C) by the J-DOSS database of Japan Oceanographic Data Center (JODC), whereas the temperature of the experimental pond at Awaji Island was 23.9–34.9 °C (av. 29.0 °C), and the seawater pond at Ishigaki Island was 25.3–31.7 °C (av. 27.0 °C), and 19.3–26.2 °C (av. 22.2 °C) in our field experiment. Therefore, we believe that comparable biomass productivity is to be expected using the ocean surface, as in our experiments.

We did not pretreat the seawater when we prepared our modified seawater-based media, although previous studies using seawater for media pretreated the seawater with NaHCO_3_ to precipitate the divalent cations Ca^2+^ and Mg^2+^ (Faucher et al. 1979; Leema et al. 2010). In our experiment, *A. platensis* NIES-39 could grow in the diluted seawater with the addition of nutrients. By using the modified seawater medium in place of SOT medium, it was possible to reduce the cost of medium to a one fifth.

The biomass productivities of *A. platensis* NIES-39 grown with four different seawater-based media and SOT medium were evaluated during four culture periods. Growth in modified seawater media was significantly lower than that in SOT medium. The pH of the seawater-based media was approximately 9 (Fig. 6b). According to previous studies, *A. platensis* shows high photosynthetic activity between pH 8.3 and 10.5 in a medium containing about 200 mM Na^+^ and shows high Na dependency of oxygen evolution at high pH (Schlesinger et al. 1996; Pogoryelov et al. 2003). Generally, the sodium concentration of seawater is about 500 mM. Therefore, the growth rate of *A. platensis* NIES-39 cultured in 1/2 and 1/3 SW media might have been higher than that in 1/4 SW media (Fig. 6a).

Although growth in modified seawater media was slow, the dry weight biomass of the harvested algae was comparable to that grown in SOT medium (Table 2). This may be explained that the algal cells grown in modified seawater-based media were heavier than those in SOT medium. The glycogen content of *A. platensis* NIES-39 grown in modified seawater media was higher than in SOT medium (Table 2). Also, according to Pogoryelov et al. (2003), *A. platensis* grown in seawater had higher carbohydrate content than in Zarrouk’s medium, which is a standard mineral medium and similar to SOT medium. It is known that as an osmotic response to the higher salinities in the media, *A. platensis* accumulates organic osmolytes such as low-molecular-weight carbohydrates, glucosyl-glycerol, and trehalose (Reed et al. 1984; Vonshak et al. 1988). Dry weights of *A. platensis* NIES-39 grown in modified seawater media might have been greater than in SOT medium due to increased contents of glycogen and other osmolytes. Moreover, it might have caused rapid self-flocculation of *A. platensis* NIES-39 in modified seawater media (Fig. 7). The relationships between morphological changes of *Arthrospira* trichomes and NaCl concentration have been reported previously (Jeeji Bai 1985; Lewin 1980; Dhiab et al. 2007; Pogoryelov et al. 2003). In response to the increase of NaCl concentration, the shape of the trichomes of *Arthrospira* is changed to straight from the helical form. Also, under solar UV radiation, the helical coiling of trichomes of *Arthrospira* becomes looser (Wu et al. 2005). Our results are consistent with these previous reports that there are some differences in the degree of helicity of *A. platensis* NIES-39 grown with different seawater media (Fig. 8). However, these results are not only caused by the difference in Na^+^ concentration in the growth media. The Na^+^ concentration of SOT medium is about 250 mM, equivalent to the Na^+^ concentration of 1/2 SW media. The change in helicity might have been caused by the difference in various elements, in particular the divalent cations Ca^2+^ and Mg^2+^.
